# Understanding and managing pterygium

**Published:** 2017-02-10

**Authors:** Anthony Bennett Hall

**Affiliations:** 1 Consultant Ophthalmologist: Hunter Eye Surgeons, Newcastle Eye Hospital, Newcastle, Australia.

A pterygium is a wing-shaped fibrovascular proliferation of the conjunctiva that grows across the cornea.^1^ Pterygium occurs more frequently in people who live in areas with high ultraviolet radiation. Dusty, hot, dry, windy, and smoky environments also play a part.^2^ Most occur on the nasal side.

## Diagnosis

### Step 1. Taking a detailed history

How long has the growth been present? Typically, this would be for many months or years. This helps to differentiate it from ocular surface squamous neoplasia (OSSN), which tends to have a shorter history (see pages 52–53).

Ask the patient if it has been getting bigger. Some pterygia are inactive and have not grown for decades.

What symptoms is the patient complaining of? There may be redness, irritation, blurring of vision, double vision, itching, and a concern about the cosmetic appearance.^3^

### Step 2: Examination

Check the visual acuity. You should always do a complete eye examination and look for other causes of discomfort or vision loss.

Measure the size of the pterygium from the limbus to the apex of the pterygium on the cornea. Record this on a diagram in the clinical record so that, the next time you see the patient, you can tell if the pterygium has grown.

Look for any atypical features that might make you worry about dysplasia (early-stage cancer), such as leucoplakia (an elevated, white, dry-looking patch), a raised gelatinous mass, oralarge, prominent feeder blood vessel. Be especially alert if you live in Africa where there is a high prevalence of OSSN.^4^

Examine the eye movements to look for any evidence of restricted movement caused by the pterygium.

Retinoscopy will reveal any with-the-rule astigmatism that may be caused by the pterygium. Corneal topography can be valuable in detecting irregular astigmatism and distortion caused or induced by pterygium.

**‘You should always do a complete eye examination and look for other causes of discomfort or vision loss.’**

**Figure:**
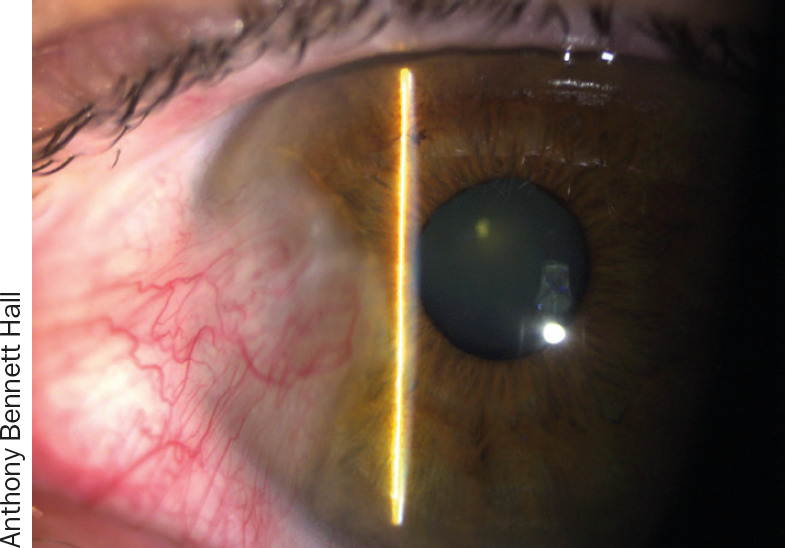
Pterygium examined using a slit lamp

### When to treat

The most important indications for treatment are:

- Involvement of, or threat to, the visual axis
- Loss of vision from astigmatism
- Restriction of eye movement
- Atypical appearance suggesting dysplasia
- Increasing size (documented by an ophthalmologist)

Less important indications are:

- Increasing size (reported by the patient)
- Symptoms of irritation and complaints of redness, etc.
- Cosmetic issues

## Counselling patients

Patients benefit from counselling before and after the operation.

Not every pterygium needs to be operated on. Some patients may expect to have their pterygium removed when simple conservative treatments such as lubricating drops or steroids may be all that is needed. It is important to explain to patients that there is a chance of recurrence, so the pterygium may come back even if it has been surgically removed. However, surgery with a conjunctival graft (as described opposite) substantially reduces the risk of recurrence.

Compile a list of indications to suit your setting. Use the list to counsel patients about their suitability for an operation. Review them in a few months to see if the symptoms have improved with conservative treatment and to check if the pterygium has grown.

Use an information leaflet to help you to counsel patients. We use a leaflet which has a picture of a pterygium, a list of indications, a description of the procedure, what to expect in the postoperative period, possible complications, and the likelihood of recurrence. The picture is useful in helping you to explain the diagnosis, the indications for surgery and the pterygium operation. Warn patients that the eye may be quite painful fora day or two.

## Complications

Patients need to be fully informed about possible complications before you start.

Complications can occur during the operation or may present later.

Intraoperative complications include:

- Perforation of the globe
- Thinning of sciera or cornea from dissection
- Intraoperative bleeding
- Excessive cautery
- Muscle damage
- Reversing the conjunctival autograft (placing it epithelial surface down)

Early postoperative complications include:

- Persistent epithelial defects Dellen formation (an area of corneal thinning adjacent to limbal swelling that prevents normal wetting of the corneal surface)
- Haematoma beneath the graft
- Loss of the graft
- Pyogenic granuloma

Late complications include:

- Recurrence
- Corneo-scleral necrosis
- Scleritis
- Endophthalmitis

Recurrence is a major late complication. The highest rate of recurrence occurs in the bare sciera technique.^1–5^ The section opposite describes a technique of excision with **conjunctival autografting**, which reduces the recurrence rate.^1^ You may wish to consider using adjuvants such as 5-fluorouracil or mitomycin C, but be aware that mitomycin C is associated with a higher rate of visually threatening complications. Adjuvants can be reserved for recurrent cases.^1^
