# Supplementary material for: Understanding and managing pterygium
Source: Community Eye Health. 2017 Feb 10;29(95):54–6. (PMC5340105)
Supplement: Related texts

# Brief report: Pterygium surgery: the conjunctival autografting technique

**Published:** 2016

## Before the operation

Consider using steroids for a few days preoperatively to reduce inflammation.

Before you begin giving the anaesthetic, check the notes to make sure you are proceeding on the correct eye. Mark the eye, as you would for any eye procedure, to avoid possible confusion.

Give the patient topical anaesthetic drops before they come into the theatre. Dilating drops will help reduce the pain from postoperative ciliary spasm.^5^

## Anaesthesia

If you have a cooperative patient, you can infiltrate local anaesthetic under the conjunctiva using a fine-gauge needle. Use a long-acting anaesthetic such as bupivacaine as this can give some hours of pain relief after the operation. Adrenaline will aid haemostasis.

Infiltrate the anaesthetic under the pterygium and under the conjunctival epithelium supero-temporally. The advantage of local infiltration is that the patient retains the ability to move the eye and can be asked to look left, right or down to expose the part of the eye that is being operated on.

Give a sub-Tenon's anaesthetic if the patient is likely to be uncooperative or if you anticipate a lengthy procedure.

You will need to reassure the patient and explain each step as you proceed with the anaesthesia and the excision.

## Pterygium excision and autoconjunctival graft

Pterygium surgery should not be delegated to the most junior trainee surgeon. Supervision of trainees should be continued until they are competent at all the steps required. This will reduce recurrence rates.^3^

Prepare the patient as you would for intraocular surgery. Wear a sterile gown and gloves, disinfect the skin around the eye and the conjunctival sac with 5% (aqueous) povidone iodine solution, and drape the patient. A scrub nurse should assist you. A surgical pack containing an eyelid speculum, two pairs of Moorfield's forceps, fine-toothed forceps, Wescott scissors, needle holder, crescent blade or No. 15 blade, bipolar or ball cautery, fine absorbable suture (7-0 or 9-0) or 10-0 nylon and swabs.

Even if you have given a sub-Tenon's block, injecting anaesthetic with adrenaline under the conjunctiva will help to elevate the pterygium off the sciera and separate the conjunctival epithelium from the underlying Tenon's capsule. The vasoconstrictive effect will also limit bleeding. A traction suture may be needed to move the eye if the patient has had a block. This may be inserted through the superior peri-limbal conjunctival tissues or be a corneal traction suture.

**Figure 1.** Dissecting pterygium off the limbus

**Figure 2.** Dissecting thin graft off Tenon's capsule

**Figure 3.** Suturing limbal corner of graft to sciera

## Excising the pterygium

To get a good view, ask the patient to look in the direction away from the pterygium. Start the excision of the pterygium by grasping it with Moorfields forceps and making radial incisions with Wescott scissors along the edges. Find the plane under the pterygium and Tenon's capsule anterior to the medial rectus muscle. Take care to stay away from the medial rectus muscle so that it is not cut or damaged inadvertently. Cut along the base of the pterygium (parallel to the limbus). Make sure you stay anterior to the plica. The pterygium should lift easily off the sciera. It becomes adherent at the limbus and you will need to use a crescent blade or No. 15 blade to carefully dissect it off the cornea (Figure 1). The sciera must be clean of any Tenon's capsule.

Ask your assistant to keep the field free of blood so that you have a clear view of the depth of your dissection. Most bleeding will stop of its own accord. Only use cautery if the bleeding is so profuse that it is likely to form a large haematoma and lift the conjunctival graft off the sciera. A little blood will act as autologous fibrin glue.

## Taking the conjunctival autograft

Ask the patient to look down. Marking the epithelium with a sterile skin marker will help you to identify the surface of the graft. Make two radial incisions in the superior

bulbar conjunctiva. The incisions should outline an area that is about the same in size as the nasal conjunctival defect. Carefully dissect the conjunctiva off the underlying Tenon's capsule (Figure 2). Once you are in the correct plane you should incise the conjunctival graft along its posterior edge. Lift the posterior edge and carefully dissect off any adherent Tenon's capsule. Your assistant may hold one corner of the graft for you. The graft may be placed epithelium up on a paper template (suture cover) before it is cut off from the limbus. This improves the handling and orientation of the thin conjunctival tissue.^2^

## Placing and suturing the graft

Orientate the graft with the limbal donor edge closest to the nasal limbus.

Fibrin glue can speed up pterygium surgery and may reduce postoperative pain.^2^ However, the cost of fibrin glue is prohibitive, even in some high-resource settings. A good alternative is 9-0 or 10-0 nylon: it is widely available, cheap, and causes no tissue reaction.^5^

Anchor the two limbal corners to the sciera to avoid posterior migration of the graft (Figure 3). Suture the remaining corners of the graft to the nasal conjunctiva. If you are using nylon, use a mattress suture to bury the knots. Place additional sutures as required to close any gaps between the graft and the nasal conjunctiva.

**Figure 4.** Graft one week after surgery

Apply chloramphenicol ointment to the conjunctiva and firmly pad the eye.

## Postoperative care

The patient will need good pain relief after surgery. We prescribe a combination of paracetamol and codeine for a day or two.

Ask the patient to instil steroid and antibiotic drops 4 times a day for a week. The topical steroid should continue for at least a month.

Examine the patient the next day to make sure that the graft is in place.

The next visit is at 1 week (Figure 4). Review the patient at 1 month and 3 months to make sure there are no complications. Signs and symptoms of recurrence usually occur 4-6 weeks after surgery.^5^

Encourage the patient to return in a year so that you can check for any recurrence of the pterygium.

## References

[1] KaufmanSCJacobsDSLeeWBDengSXRosenblattMIShteinRM. Options and adjuvants in surgery for pterygium: a report by the American Academy of Ophthalmology. Ophthalmol 2013;120(1):201–8. Epub 2012/10/16.10.1016/j.ophtha.2012.06.06623062647

[2] KoranyiGSeregardSKoppED. Cut and paste: a no suture, small incision approach to pterygium surgery. Br J Ophthalmol. 2004;88(7):911–4. Epub 2004/06/19.1520523610.1136/bjo.2003.032854PMC1772242

[3] HirstLW. The treatment of pterygium. Surv Ophthalmol. 2003;48(2):145–80. Epub 2003/04/11.1268630210.1016/s0039-6257(02)00463-0

[4] GichuhiSSagooMSWeissHABurtonMJ. Epidemiology of ocular surface squamous neoplasia in Africa. Trop Med Int Health. 2013;18(12):1424–43. Epub 2013/11/19.2423778410.1111/tmi.12203PMC4440345

[5] SheppardJDMansurAComstockTLHovanesianJA. An update on the surgical management of pterygium and the role of loteprednol etabonate ointment. Clin Ophthalmol. 2014;8:1105–18. Epub 2014/06/27.2496666410.2147/OPTH.S55259PMC4063821
